# Supplementary material for: Deep Learning for Image-Based Cassava Disease Detection
Source: Front Plant Sci. 2017 Oct 27;8:1852. doi: 10.3389/fpls.2017.01852 (PMC5663696; doi:10.3389/fpls.2017.01852)
Supplement: Supplementary file 1 [file DataSheet1.DOCX]

Supplementary Data: 6 figures. 2 tables.


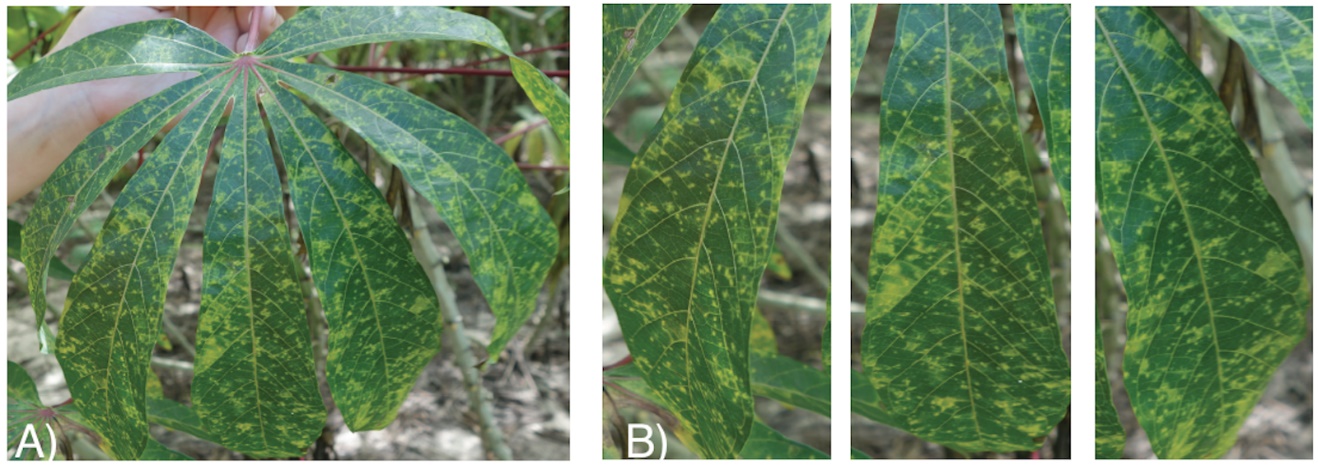


Figure S1. Image of cassava leaf infected with cassava brown streak disease from the A) original dataset and B) leaflet dataset


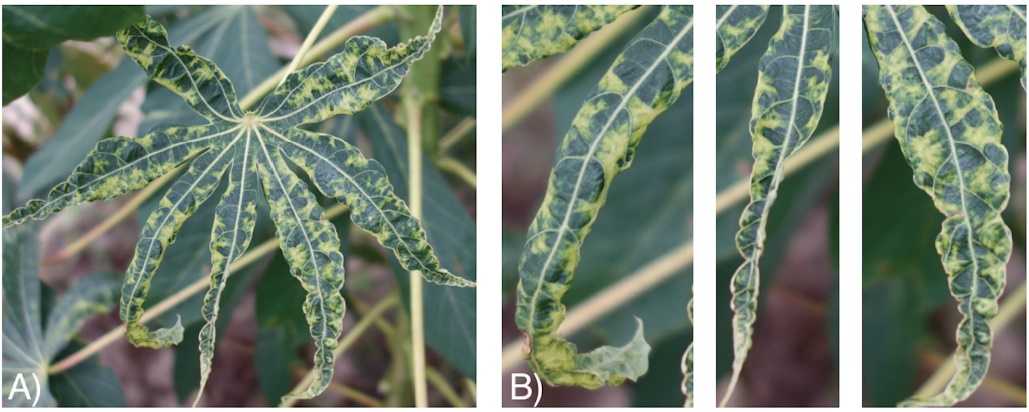


Figure S2. Image of cassava leaf infected with cassava mosaic disease from the A) original dataset and B) leaflet dataset


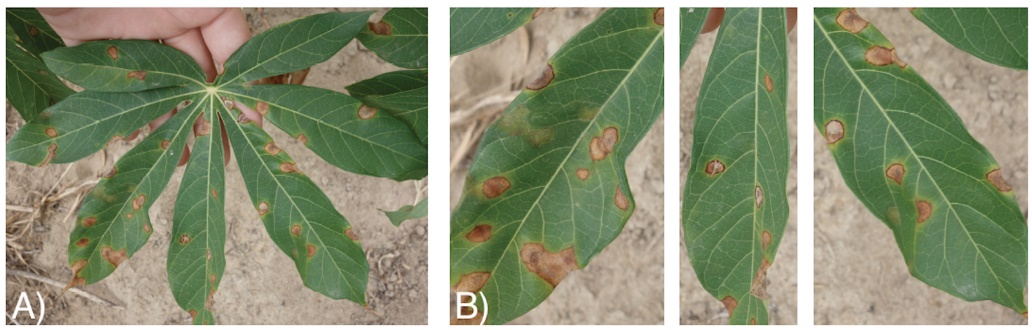


Figure S3. Image of cassava leaf infected with cassava brown leaf spot from the A) original dataset and B) leaflet dataset


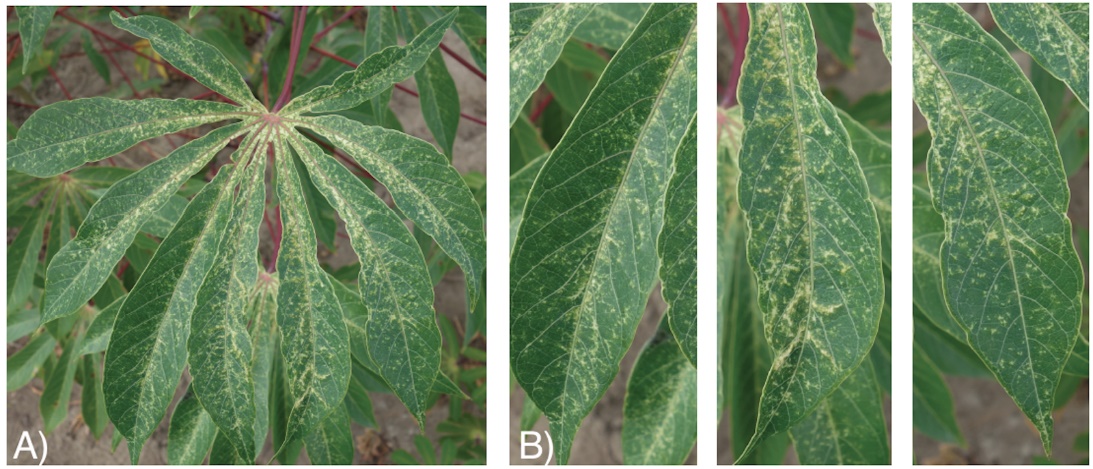


Figure S4. Image of cassava leaf affected by green mite damage from the A) original dataset and B) leaflet dataset


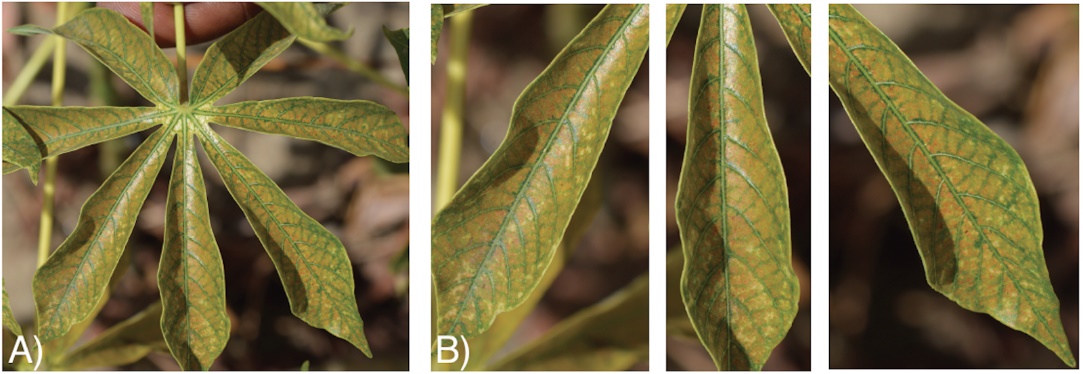


Figure S5. Image of cassava leaf affected by red mite damage from the A) original dataset and B) leaflet dataset.

| Class | Variety | Age (months) |
| --- | --- | --- |
| Healthy | Alberty | 5 |
|  | Mkuranga 1 | 5 |
|  | Shibe | 4 |
|  | Tajirika | 4 |
|  | TZ130 | 4 |
|  | Yizaso | 4 |
| Cassava Brown Streak Virus | Alberty | 5 and 8 |
|  | Mkuranga 1 | 5 |
|  | Kikombe | 5 and 8 |
|  | Mkuranga1xKiroba | 4 |
| CMD | Cheroke | 4 |
|  | Kikombe | 4 |
|  | Kipusa | 4 |
| Green Mite Damage | Alberty | 4 |
|  | CH005-2003 | 4 |
| Red Mite Damage | CH005-2003 | 4 |
|  | Kipusa | 4 |
|  | Nase 14 | 4 |
|  | Shibe | 4 |
| Brown Leaf Spot | Cheroke | 4 |

Table S1. Description of varieties and plant age photographed in each class.

|  | | **Inception** | **Knn** | **SVM** |
| --- | --- | --- | --- | --- |
| **Train:20% Test:70%** | **Original** | 83.5 | 72.2 | 84.4 |
|  | **Leaflet** | 89.1 | 78.6 | 90.0 |
| **Train:40% Test:50%** | **Original** | 86.0 | 76.5 | 88.2 |
|  | **Leaflet** | 90.9 | 81.0 | 92.0 |
| **Train:50% Test:40%** | **Original** | 86.1 | 77.7 | 88.0 |
|  | **Leaflet** | 91.9 | 81.8 | 92.1 |
| **Train:60% Test:30%** | **Original** | 88.3 | **80.1** | 89.4 |
|  | **Leaflet** | 92.2 | 83.2 | 92.8 |
| **Train:80% Test:10%** | **Original** | **89.6** | 78.4 | **90.9** |
|  | **Leaflet** | **92.9** | **83.3** | **93.0** |

Table S2. Overall accuracy for transfer learning using three machine learning methods.


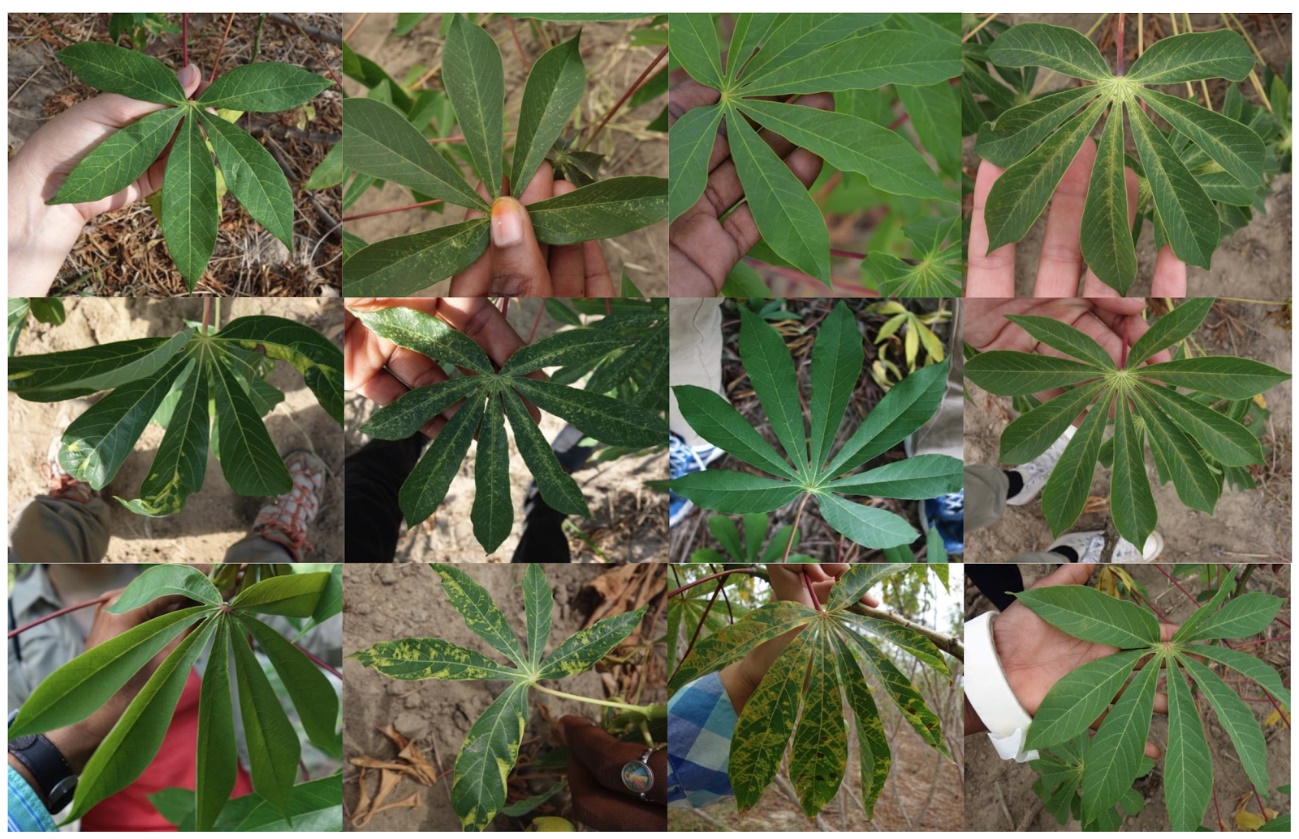


Figure S6. Representation of various background objects captured in the images.
